# Experimental Study on Rapeseed Drying Characteristics with Magnesium Sulfate as Solid Desiccant

**DOI:** 10.3390/molecules30173604

**Published:** 2025-09-03

**Authors:** Elena V. Fomenko, Natalia N. Anshits, Galina V. Akimochkina, Timur Yu. Ivanenko, Evgeny V. Morozov, Vladimir V. Yumashev, Leonid A. Solovyov, Nikolay P. Shestakov, Vasily F. Shabanov

**Affiliations:** 1 Institute of Chemistry and Chemical Technology, Federal Research Center “Krasnoyarsk Science Center of the Siberian Branch of the Russian Academy of Sciences”, Akademgorodok 50/24, 660036 Krasnoyarsk, Russia; anshitsnn@mail.ru (N.N.A.); agv3107@mail.ru (G.V.A.); timivonk@gmail.com (T.Y.I.); morozovev@iph.krasn.ru (E.V.M.); yumashev_vlad@mail.ru (V.V.Y.); leosol@icct.ru (L.A.S.); 2 Federal Research Center “Krasnoyarsk Science Center of the Siberian Branch of the Russian Academy of Sciences”, Akademgorodok 50, 660036 Krasnoyarsk, Russia; shabanov@ksc.krasn.ru; 3 Kirensky Institute of Physics, Federal Research Center “Krasnoyarsk Science Center of the Siberian Branch of the Russian Academy of Sciences”, Akademgorodok 50/38, 660036 Krasnoyarsk, Russia; nico@iph.krasn.ru

**Keywords:** sorption drying, rapeseed, desiccant, magnesium sulfate, moisture content

## Abstract

Rapeseed is a valuable oilseed crop, and efficient drying plays a crucial role in preserving its quality. Because of the high moisture content in rapeseed, drying using the conventional methods may cause it to overheat. The benefit of energy-efficient sorption drying is that it allows one to carefully remove moisture from seeds without using heat, thus ensuring better quality. This study focuses on the characteristics of rapeseed drying using fine crystalline magnesium sulfate MgSO_4_·*n*H_2_O as a desiccant. The properties of the desiccant were analyzed using the SEM–EDS, XRD, ATR–MIR, and DSC-TG techniques before and after contacting rapeseed. The findings demonstrate that the desired moisture content of 7–8% can be achieved within 60–240 min, depending on the initial moisture content of rapeseed (ranging from 12% to 16%) and the desiccant-to-rapeseed ratio (1:2, 1:4, or 1:6). An analysis of crystalline hydrates after sorption drying indicates that the desiccant can be reused without intermediate regeneration during multi-stage drying of two to three rapeseed batches. The germination capacity of the seeds after sorption drying was as high as 90%, meeting the standards for elite rapeseed categories. This research demonstrates that sorption drying using magnesium sulfate is an efficient method for reducing moisture content in oilseeds, while maintaining their quality.

## 1. Introduction

Rapeseed (*Brássica nápus*) is one of the most important oilseed crops belonging to the *Brassicaceae* family, a source of vegetable oil and valuable high-protein ingredients. The high demand for rapeseeds is driven by the versatility of this crop as rapeseed oil can be used in food, pharmaceutical, cosmetic, chemical, textile, leather, soap-making, and dyeing industries. The growing demand for rapeseed oil is met by the feasibility of using it as an environmentally friendly and renewable fuel source [1,2,3].

As interest in rapeseed continues to grow, the reduction in post-harvest losses provides a significant opportunity to increase their availability and production volume [4]. According to most studies and reports [4,5,6,7,8,9,10], global losses for various agricultural products range from 15% to 60%, depending on the region. The analysis of various post-harvest losses and the main factors responsible for these losses are discussed in detail in refs. [4,5,9]. For example, various microbial pests can cause damage to products after harvest [10]. It is noted [5] that oilseed varieties with high oil content gain special attention, as due to high moisture content vegetable oil can degrade, leading to deterioration. The average loss rate for oil crops is about 20% [6,11]. In the case of adverse weather conditions during harvesting, losses can reach up to 40% [12].

Drying is a common technique for improving the product stability. Because of uneven crop maturation, rapeseeds of heterogeneous quality have an initial moisture content of 15–25%, whereas the standardized moisture content is expected to be 7–8% [13]. The critical moisture content of rapeseed, at which the so-called free moisture appears in seeds and all vital processes are abruptly sped up (leading to active respiration and increasing the risk of microbial development, potentially resulting in deterioration of quality and seed germination), is 8.3–8.6% [14].

The critical moisture content varies across different crops and is dependent on chemical composition of seeds. For oilseed crops such as rapeseed, it is lower than for cereals and legumes because of the high content of fat in oilseeds, which is characterized by lower moisture retaining capacity compared to proteins and starch in cereals. Rapeseed drying is based on modern drying theory [15,16], but it also has its own unique characteristics. These characteristics are determined by the physico-mechanical, physico-chemical and biochemical properties of rapeseed, which make it a challenging object for drying. Because of their high moisture and oil contents, rapeseeds are more prone to oxidative changes and microbial spoilage than cereals [6]. Therefore, high-quality, efficient and prompt drying is crucial for preserving the germination and processing characteristics of rapeseed.

Seed batches of any crop are dried under milder conditions than seeds intended for food use [17,18,19]. Compared to cereals, drying of thermolabile oilseeds is a more complex and expensive process that has specific features. First, the oil and protein contents in moist rapeseeds are high, so they pose a significant risk of spontaneous ignition if wrong temperature regimes are used. Second, rapeseed is highly sensitive to elevated temperatures. Exposure to heat induces prominent physicochemical and biochemical changes in rapeseeds (e.g., accelerated oil rancidity and protein denaturation).

Hot-air drying of rapeseed in conventional grain dryers is challenging because of the risk of overheating as well as the higher rapeseed layer resistance compared to that of wheat grain. Rapeseed is a small-seeded crop, so greater air pressure needs to be applied to overcome layer resistance during drying agent passage, resulting in a shallower filling depth compared to cereal grains when using silos of the same diameter and ultimately reducing the overall drying efficiency. The high temperature of a drying agent together with long-term exposure may have a detrimental effect on commercial rapeseed. As a result, the contents of essential amino acids in rapeseed decrease, the acid degree value increases, and seeds become brittle, thus facilitating microbial colonization and accelerating oxidative processes [4,5,6,9,10,11]. Furthermore, overdried rapeseed is characterized by impaired oil separation from the press cake, leading to reduced oil yield and impairing press cake quality. Therefore, conventional hot-air drying cannot fully guarantee that seed quality is retained, and milder drying conditions are needed for rapeseed compared to cereal grains, thus reducing the throughput of industrial drying systems.

Meanwhile, it should be borne in mind that thermal drying is an energy-intensive process. In some developed countries, the energy consumed during drying makes up approximately 10–25% of the nation’s total annual industrial energy consumption [20,21]. In this regard, there is a global push to actively promote the development of energy-efficient and environmentally friendly drying technologies [22,23,24] that would primarily focus on consumption of alternative energy sources [25]. When using microwave (84%), infrared (~67%), microwave + hot air (~75%) and infrared + hot air (~54%) drying methods, energy savings were achieved compared to the convection hot air drying process [23].

The main purpose of drying oilseeds should be to reduce moisture content to the necessary level and maintain the quality of oil. Existing methods for drying rapeseed include convection dryers, such as column, drum and floor dryers, as well as ambient air drying and emerging technologies such as microwave, infrared and heat pump drying [23,26,27,28,29,30,31,32]. The latest drying methods and technologies used to bring oilseeds to a safe moisture level are described in [23]. Currently, microwave drying of rapeseed seems to be a promising technique [26,27].

The number of damaged seeds heated with microwaves is lower than that of seeds heated with hot air. Therefore, for rapeseed, Ren et al. [28] investigated the effect of microwave drying on the microstructure of rapeseed and on oil production. Geneev et al. [29] proposed a microwave drying device with a two-stage drying process, which can increase the process productivity compared to existing methods. However, seeds dried using microwaves seem to be completely sterile and cannot germinate [26]. Bulgakov et al. [30] suggested using infrared radiators to intensify the rapeseed drying process. Xu et al. [31] studied the effect of dielectric treatment on the drying characteristics of rapeseed and the physicochemical properties of cold-pressed oil.

The objective of drying seeds is to preserve their viability at the highest possible level. For seeds, the drying temperature must not exceed 43–48 °C to maintain high germination [32]. Heat pump dryers provide this temperature with low energy consumption. Increasing the energy efficiency of the process is achieved by increasing the drying intensity and reducing the moisture content of the heat carrier during the drying of seeds. A study by Snezhkin et al. [32] showed that the use of a drying unit with a heat pump can improve the quality of rapeseed compared to an installation running on electric heating.

Sorption drying allows one to reduce the moisture content in thermolabile materials, including grains and seeds, without exposure to external heat, while maintaining or even improving quality parameters [23,24,33]. This energy-efficient sorption seed drying technology relies on the use of efficient desiccants. Magnesium sulfate MgSO_4_·*n*H_2_O is a very effective desiccants that offers a number of advantages. These include its neutrality, rapid water adsorption, high water retention capacity (7 moles per 1 mole of anhydrous salt), and low regeneration temperature [34]. In particular, it has been demonstrated for wheat seeds that sorption drying using magnesium sulfate (granular kieserite form) as a desiccant is an efficient method for ensuring target moisture contents, while preserving high germination energy and viability of seeds [35]. The high water capacity of the kieserite also allows the drying of multiple batches of seeds without the need for the intermediate regeneration of the desiccant. Furthermore, after regeneration, the desiccant maintains stable sorption characteristics at a high level.

Desiccants have not been employed for rapeseed drying thus far. However, considering the current agricultural trends, application of drying agents for rapeseed drying could open up new avenues. Studies have demonstrated [35,36,37,38] that the use of specialized adsorbents can significantly accelerate moisture removal, thus improving seed quality and reducing the risk of fungal diseases. Additionally, launching these technologies could contribute to more efficient use of resources, reducing energy costs and drying time. Further research is needed to identify the optimal conditions for using desiccants to dry oilseeds. This allows the adaptation of sorption methods to new conditions and increases the rapeseed yield.

The present study focused on using magnesium sulfate MgSO_4_·*n*H_2_O as a desiccant during sorption drying of rapeseed. The aim of the study was to characterize the desiccant using a combination of experimental techniques, explore the kinetic patterns of moisture content in rapeseed upon contact with magnesium sulfate, and determine the rapeseed germination after sorption drying with MgSO_4_·*n*H_2_O.

## 2. Results and Discussion

Kieserite of fine crystalline grade, a commercial magnesium sulfate agrochemical [39], was used as a solid desiccant for sorption drying of rapeseed. The main reasons leading to this decision are the following:

1. Magnesium sulfate is a widely used inexpensive fertilizer in agriculture that does not pollute the soil. It provides essential nutrients such as sulfur and magnesium to plants and supports crop seed storage [40].
2. In an anhydrous state, magnesium sulfate is widely used as a desiccant [41]. It forms compounds with the formula MgSO_4_·*n*H_2_O, where *n* can range from 1 to 11 [42]. The predominant and most stable natural magnesium sulfates include epsomite MgSO_4_∙7H_2_O, hexahydrate MgSO_4_∙6H_2_O, and kieserite MgSO_4_∙H_2_O [43]. Previous studies have shown that contact sorption drying of wheat seeds using granular kieserite as a post-harvest treatment is an effective method that reduces moisture content in the seeds while maintaining high germination rates [35,37].
3. Magnesium sulfate is considered safe, and is used in the food industry as an additive E518 that has various functions. It can be used as a firming agent in canned vegetables and fruits, as a flavor enhancer in certain dairy products, and as a salt substitute. It also serves as a desiccant to help maintains product dryness. Magnesium sulfate is acceptable in foods conforming to the following commodity standards [44,45].

Desiccant characterization is needed to ensure drying efficiency. Desiccant characterization was conducted using particle size analysis, scanning electron microscopy–energy dispersive spectroscopy (SEM–EDS), X-ray diffraction analysis (XRD), infrared spectroscopy (ATR–MIR), and simultaneous thermal analysis (DSC–TG). The characteristics of the initial desiccant are reported in Section 2.1 and those after sorption drying of rapeseed in Section 2.4.

### 2.1. Characterization of the Initial Desiccant

**Figure 1:**
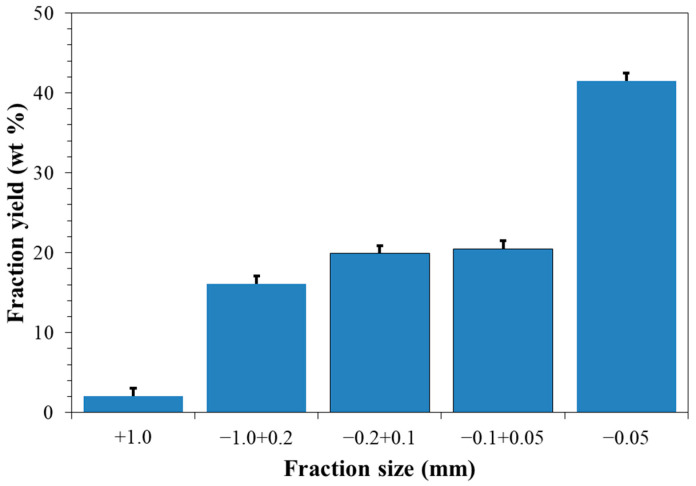
The granulometric composition of the desiccant.

Figure 1 shows the particle size distribution of the initial desiccant. Magnesium sulfate MgSO_4_·*n*H_2_O consists of 98% particles sized < 1.0 mm. There are only 2% of larger particles (+1.0 mm), with size corresponding to that of rapeseed (0.9–2.2 mm). The −1.0 mm fraction was used as a sorbent for rapeseed drying. The moisture content in the desiccant determined after calcination to constant mass at 400 °C was 24.88 wt%. This composition corresponds to the crystalline hydrate formula MgSO_4_·2.21H_2_O.

**Figure 2:**
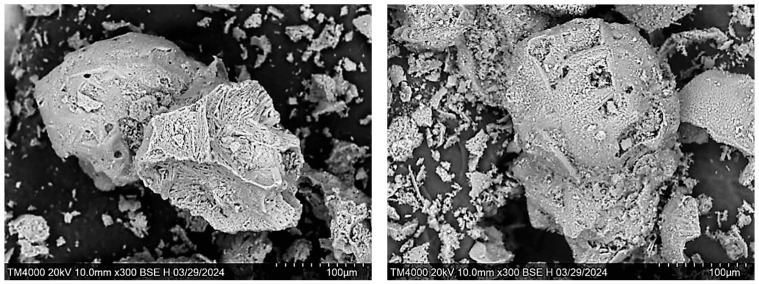
SEM image of the desiccant particles.

**Figure 3:**
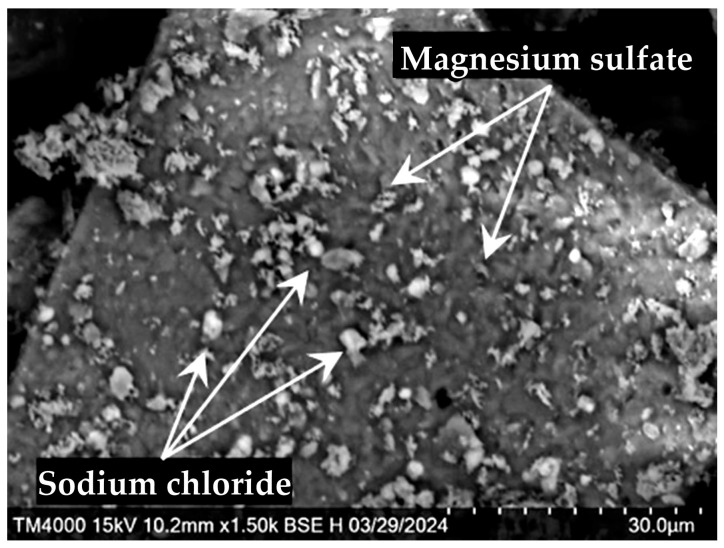
SEM image of a splinter of desiccant particle with sodium chloride crystals (white) on the surface.

**Figure 4:**
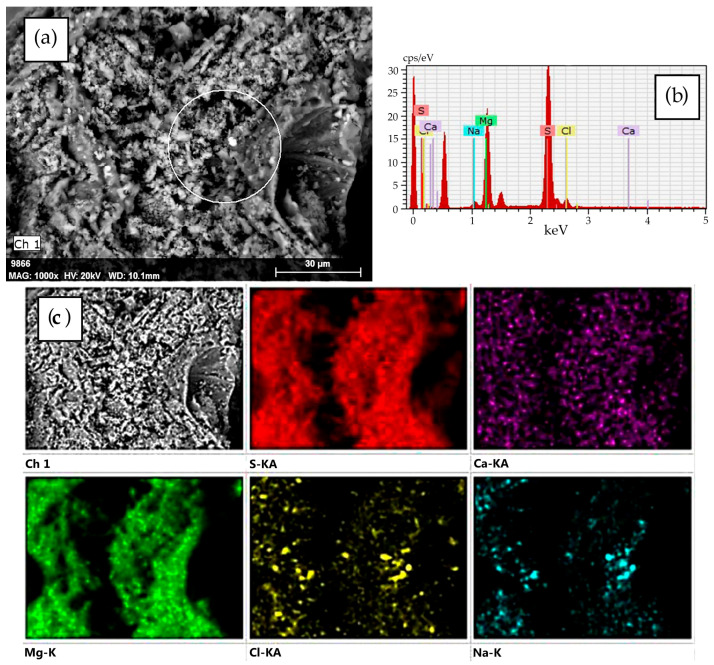
(**a**) SEM image of a desiccant particle; (**b**) the energy-dispersive X-ray spectra for the area indicated in part (**a**) (white circle); and (**c**) the element distribution map for this particle.

The SEM-EDS data demonstrate that the desiccant consists of fragmented decrystallized magnesium sulfate particles (Figure 2). Both the external and internal surfaces contain finely dispersed sodium chloride particles sized ≤ 5 μm (Figure 3). The element distribution map clearly illustrates the association of sodium and chlorine (halite) (Figure 4c). Calcium sulfate is uniformly distributed within magnesium sulfate without forming a distinct crystalline phase (Figure 4).

**Figure 5:**
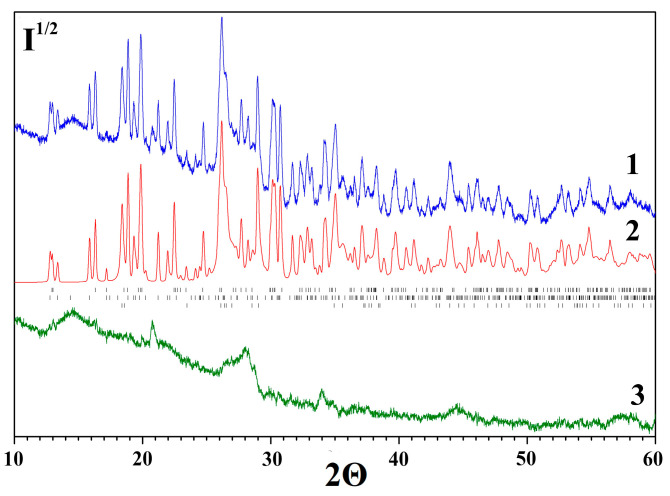
Experimental (1), calculated (2) and difference in (3) X-ray diffraction patterns of the initial desiccant. The positions of the Mg(SO_4_)·H_2_O, Mg(SO_4_)·2.5H_2_O and Mg(SO_4_)·4H_2_O peaks are marked with dashes according to the ICDD PDF database [46].

Figure 5 shows the observed and calculated X-ray diffraction patterns of the desiccant after refinement using the derivative difference minimization method. The broad halo in the difference curve within the 2θ angle range = 12–20° is attributed to the X-ray scatter from the protective film. The remaining difference modulations are caused by discrepancies between the calculated and experimental diffraction peak profiles, the X-ray amorphous component, and unaccounted trace impurities. The phase composition of the initial desiccant includes the following phases (wt%): MgSO_4_·H_2_O, 28(1); MgSO_4_·2.5H_2_O, 20.0(5); and MgSO_4_·4H_2_O, 28.2(7). Sodium chloride NaCl is also present—0.8(1) wt% [46].

### 2.2. Drying Kinetics of Rapeseed

A characterized desiccant MgSO_4_∙*n*H_2_O of a certain particle size distribution, as well as having chemical and phase compositions with a specified number of water molecules (*n* = 2.21) in the crystalline hydrate, was used to determine the sorption drying kinetics of rapeseed.

#### 2.2.1. Single-Stage Sorption Drying

**Figure 6:**
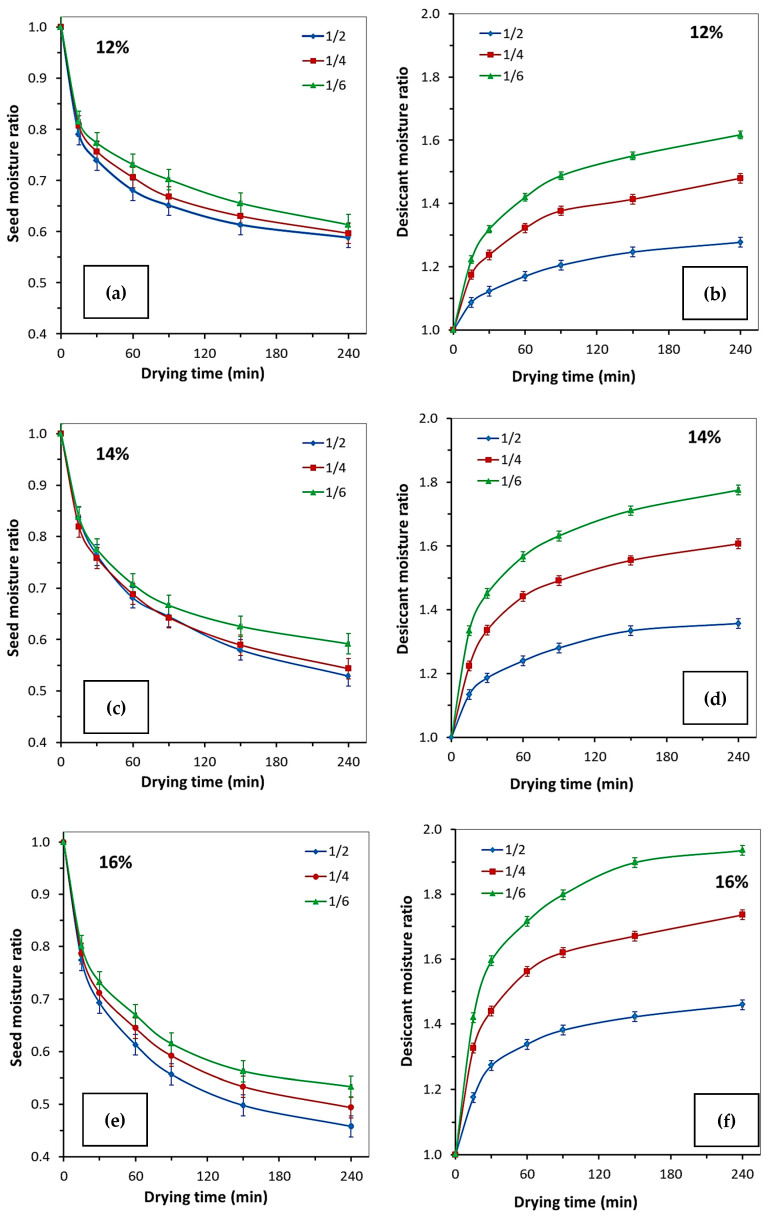
The changes in moisture ratio of rapeseed (**a**,**c**,**e**) and desiccant (**b**,**d**,**f**) during sorption drying at various desiccant-to-rapeseed mass ratios.

Table 1 and Figure 6 show the moisture contents in rapeseed and desiccant at specific time points after the initiation of sorption drying of rapeseed with different initial moisture contents (MC_0_ –12, 14, and 16 wt%) and the desiccant-to-rapeseed ratios of 1:2, 1:4, and 1:6. Data analysis demonstrates that at MC_0_ = 12 wt%, the target moisture content of rapeseed [3] of ~8 wt% is achieved after 60 min sorption drying at 1:2 and 1:4 desiccant-to-rapeseed ratios and after 90 min sorption drying at the 1:6 desiccant-to-rapeseed ratio. For rapeseed with MC_0_ = 14 wt%, the required moisture content is attained after 150 min drying at 1:2 and 1:4 ratios and after 240 min drying at a 1:6 ratio. In the case of rapeseed with MC_0_ = 16 wt%, the target moisture content is attained after 150 min sorption drying at a 1:2 ratio and after 240 min drying at a 1:4 ratio.

Hence, after the 4 h contact between rapeseed and desiccant, the moisture content decreases to ~7–8 wt% for all the investigated MC_0_ and desiccant ratios, with the only exception being that for high-moisture rapeseed (16 wt%) at a 1:6 desiccant-to-rapeseed ratio, the post-drying moisture content is 8.5 wt%.

**Figure 7:**
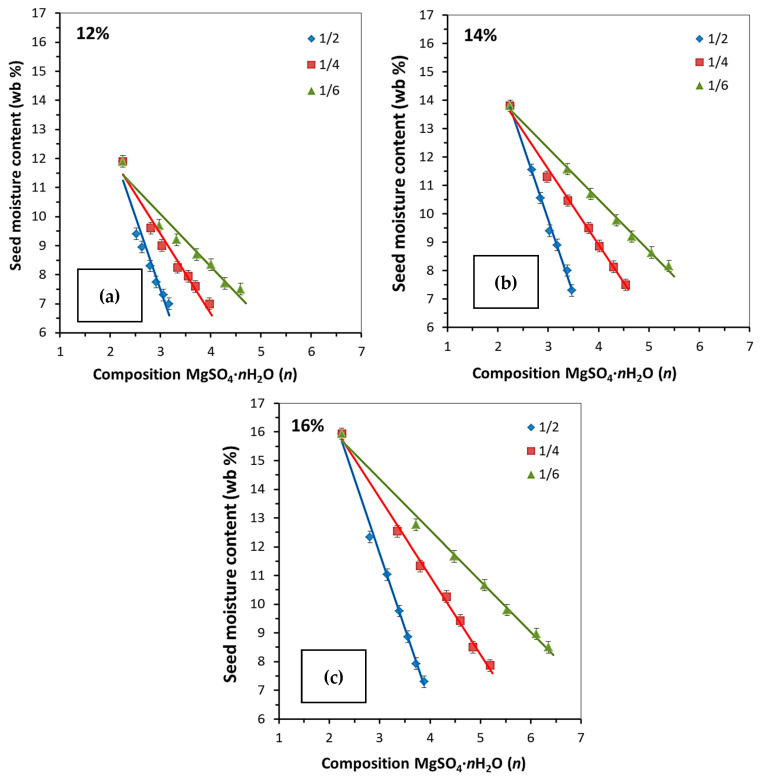
The relationship between the rapeseed moisture content and the composition of MgSO_4_·*n*H_2_O crystal hydrate during sorption drying at various desiccant-to-rapeseed mass ratios and initial rapeseed moisture content: (**a**) 12; (**b**) 14; and (**c**) 16 wt%.

The composition of crystalline hydrates after sorption drying (Table 2) indicates that the desiccant can be reused without intermediate regeneration for all the investigated desiccant-to-rapeseed ratios and MC_0_ of rapeseed. A correlation between moisture content in rapeseed and the composition of crystalline hydrates during sorption drying was revealed, being linear in all cases (Figure 7). The number of water molecules in the crystalline hydrate structure increases during drying as the initial moisture content in rapeseed rises, while the desiccant proportion in the mixture declines.

#### 2.2.2. Three-Stage Sorption Drying

Multi-stage sorption drying of rapeseed was conducted to evaluate the feasibility of reusing MgSO_4_·*n*H_2_O without intermediate regeneration. The initial moisture contents in rapeseed were ~12, 14, and 16 wt%; the desiccant-to-rapeseed mass ratio was 1:2. Three rapeseed batches were successively added to the desiccant; each drying stage lasted 4 h. Table 3 lists the moisture contents in each of the three rapeseed batches after 4 h drying, the moisture contents in the desiccant, and the number of water molecules *n*H_2_O in the MgSO_4_·*n*H_2_O crystalline hydrate.

The findings demonstrate that for rapeseed with MC_0_ = 12 wt% and MC_0_ = 14 wt%, the moisture contents in all three successively dried batches (without intermediate desiccant regeneration) ranged between 7 wt% and 8 wt%, meeting the required specifications [13]. For rapeseed with MC_0_ = 16 wt%, the target moisture content range of 7–8 wt% was achieved for the first two batches, while the moisture content of the third batch was ~10 wt%. The composition of crystalline hydrates after sorption drying (Table 3) confirms that the desiccant could be reused without intermediate regeneration when the target rapeseed moisture content was successfully achieved.

The kinetic dependences of changes in moisture content in the desiccant during three-stage sorption drying of rapeseed with different initial moisture contents were calculated using Equation (1) [17]:

MR = MC_t_/MC_0_ (1)

where MR is the moisture ratio (dimensionless); MC_0_ and MC_t_ are the initial moisture content and moisture content at drying time t, respectively, calculated on a wet basis (% wb).

**Figure 8:**
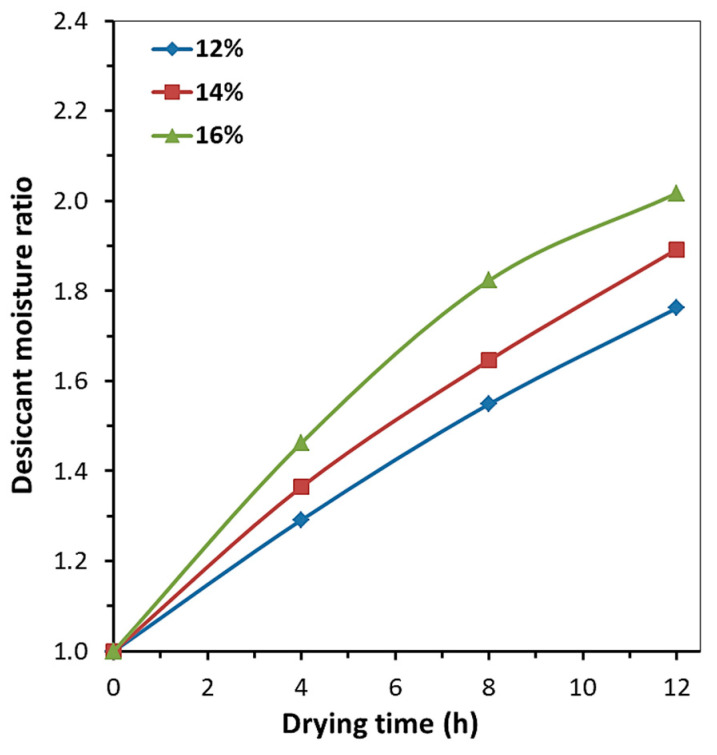
The changes in moisture ratio for desiccant during three-stage sorption drying of rapeseeds with MC_0_ 12, 14, 16 wt% at desiccant-to-rapeseed mass ratio = 1:2.

The resulting moisture saturation profiles of desiccant (Figure 8) attest to stable moisture adsorption and sufficient sorption capacity of the crystalline hydrate for sorption drying of three rapeseed batches with MC_0_ = 12 wt% and MC_0_ = 14 wt% or two batches with MC_0_ = 16 wt%.

The maximum moisture release rate was observed during the first drying stage for the first rapeseed batch (Table 3). The desiccant ensured consistent moisture release regardless of the initial moisture content in seeds until reaching crystalline hydrate compositions ranging from MgSO_4_·3.20H_2_O to MgSO_4_·3.88H_2_O. For further rapeseed batches with moisture contents of 12 wt% and 14 wt% added to the desiccant at the second and third drying stages, the moisture release rate decreased monotonically, although having no effect on process efficiency and ensuring attainment of target moisture content in seeds. Moisture contents in the desiccant after drying of three rapeseed batches with moisture contents of 12 and 14 wt% correspond to the compositions of crystalline hydrates MgSO_4_·5.29H_2_O and MgSO_4_·6.04H_2_O, respectively (Table 3). At MC_0_ = 16 wt% in rapeseed, the moisture content in the desiccant after drying of the second batch corresponds to the composition of crystalline hydrate MgSO_4_·5.65H_2_O; after that, the moisture release rate decreases noticeably at the third stage (Table 3).

Hence, the kinetic dependences of sorption drying of rapeseed with initial moisture contents of 12, 14, and 16 wt% were obtained using the agrochemical MgSO_4_·*n*H_2_O at a desiccant-to-rapeseed ratios of 1:2, 1:4, and 1:6. The moisture content in the desiccant and the compositions of crystalline hydrates were determined. The study demonstrates that the desiccant can be reused during multi-stage sorption drying of three successively added rapeseed batches with the initial moisture content of 12–14 wt% and two batches with 16 wt% moisture content at a 1:2 desiccant-to-rapeseed mass ratio. The compositions of magnesium sulfate crystalline hydrates prove that the desiccant can be reused without intermediate regeneration.

### 2.3. Rapeseed Germination

It is crucial that the permissible heating temperatures are not exceeded during rapeseed drying in order to avoid protein damage and preserve seed viability. The recommended maximum heating temperature for seed-grade rapeseed is 45 °C. Seeds are allowed to be heated in floor dryer systems to temperatures not exceeding +30–35 °C. An alternating drying regime, where cold airflows are alternated with warm ones, is used to preserve germination qualities. During thermal drying, the seeds need to be mixed vigorously to prevent local overheating hotspots. Heating of the seed embryo to 50–60 °C results in complete loss of viability. After several months of storage, these rapeseeds can be used only for producing low-quality oil.

The temperature of the mixture was measured to prevent rapeseed from heating upon contacting magnesium sulfate because of crystalline hydrate formation. The laboratory temperature was taken into account to properly compare the experimental data on thermal heating. When sorption drying of rapeseeds from the initial MC_0_ of 12, 14, and 16 wt% to the final MC_240_ of ~8 wt% occurs, the maximum heating value is observed after sorption drying for 1 h (5, 7 and 9 °C for different initial moisture contents, respectively). These values indicate that the permissible safe heating of rapeseeds contacting the desiccant will not be exceeded.

Determining rapeseed germination [47,48] after sorption drying from the initial MC_0_ of 12, 14, and 16 wt% to the final MC_240_ of ~8% showed that the seed germination capacity was 92 ± 2%, 91 ± 3%, and 89 ± 1%, respectively. According to the requirements specified in ref. [49], seedling quality for the original and elite rapeseed categories needs to be at least 85%, which is exceeded by the germination values obtained in the experiments in this study. These findings confirm that the contact between desiccant and rapeseeds during drying is considered safe for seed germination.

### 2.4. Characterization of Desiccant After Sorption Drying of Rapeseed

**Figure 9:**
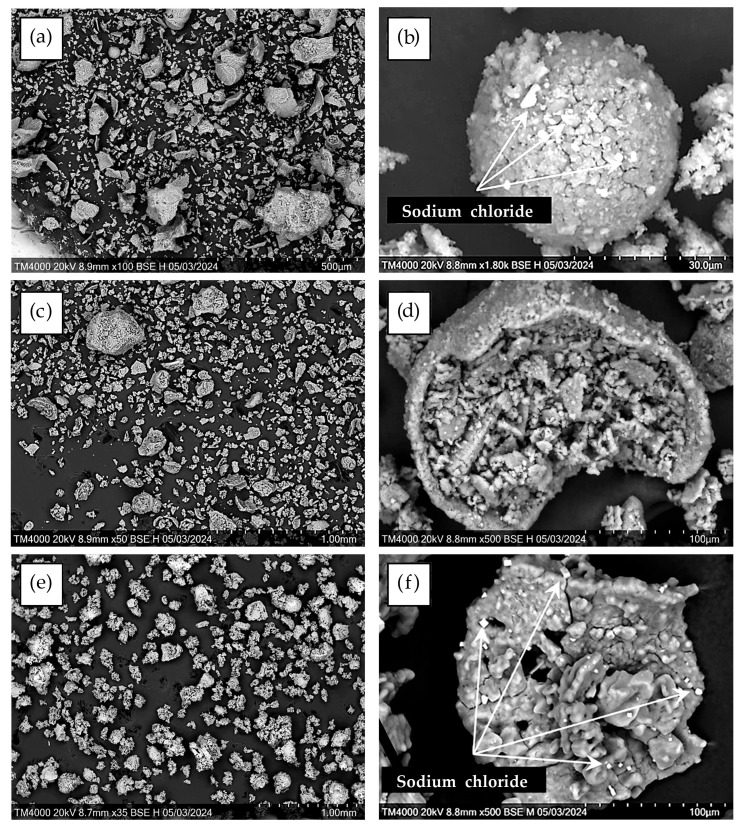
SEM image of desiccant particles after three-stage sorption drying of rapeseed: (**a**,**b**) stage I; (**c**,**d**) stage II; and (**e**,**f**) stage III.

The SEM–EDS data suggest (Figure 9) that the desiccant particle size goes down from 500 μm to 5–20 μm with each successive cycle of sorption drying of rapeseed, while the number of fractured particles increases. Finer particles form loose aggregates that densely fill cavities of large fragments of spheroidal particles and are firmly anchored within them (Figure 9d). The particle surface is extensively cracked. Calcium, present at trace amounts (total content < 0.2%), is associated with magnesium sulfate throughout its volume. Like in the initial desiccant, sodium chloride (1.0–2.5%) remains the major impurity (Figure 3 and Figure 4), with its crystals preferentially residing on the surface of magnesium sulfate particles.

**Figure 10:**
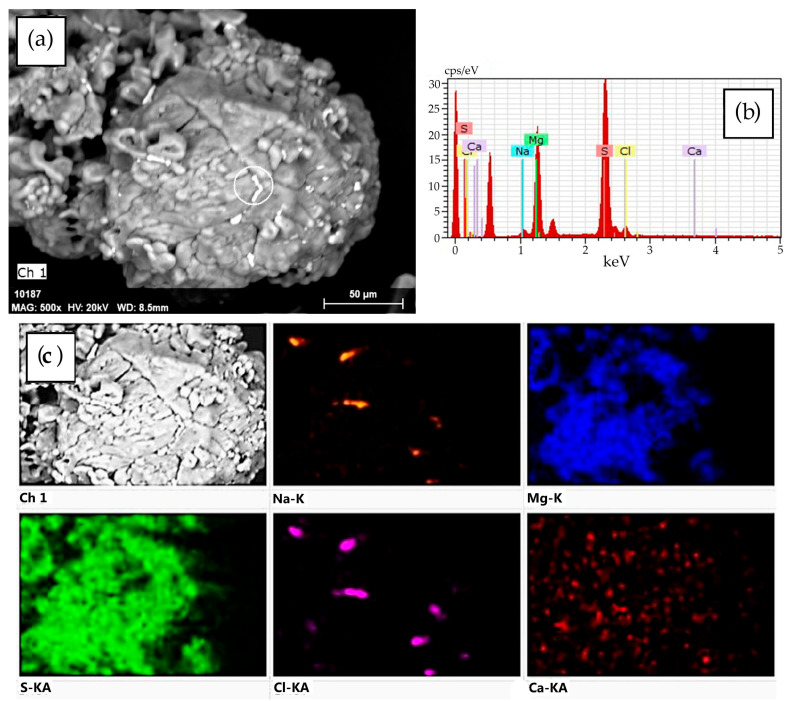
(**a**) SEM image of a desiccant particle after stage III sorption drying of rapeseed; (**b**) the energy-dispersive spectra for the area indicated in part (**a**) (white circle); and (**c**) the elements distribution map for this particle.

The size of halite crystallites increases with each subsequent drying cycle; linear sodium chloride inclusions sized up to 7 μm become observed after the third cycle (Figure 10), predominantly residing along particle surface cracks.

**Figure 11:**
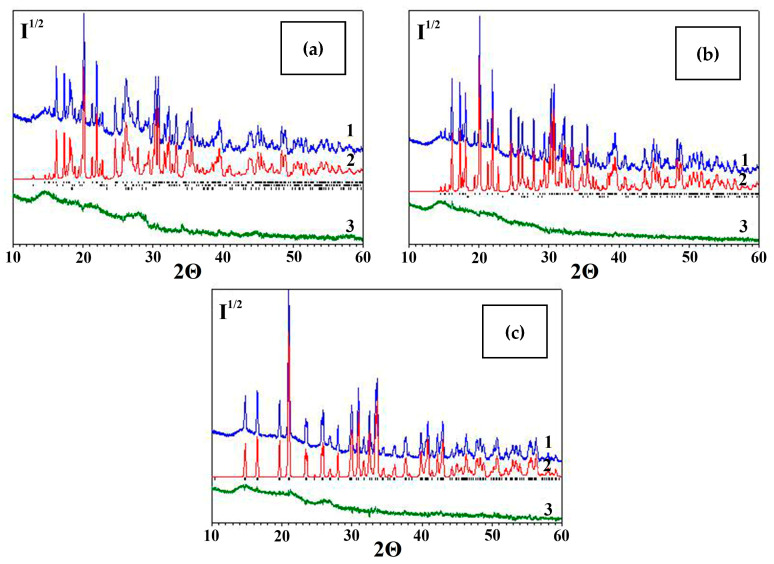
X-ray diffraction patterns of the desiccant after three-stage sorption drying of rapeseed: (1) the experimental one; (2) the spectrum calculated using the DDM method; and (3) the difference spectrum. The positions of the MgSO_4_·*n*H_2_O peaks are marked with dashes according to the ICDD PDF database [46]: (**a**) stage I (MgSO_4_·H_2_O, MgSO_4_·4H_2_O and MgSO_4_·6H_2_O); (**b**) stage II (MgSO_4_·H_2_O and MgSO_4_·6H_2_O); and (**c**) stage III (MgSO_4_·7H_2_O).

The phase compositions of magnesium sulfate after each of the three cycles of multi-stage rapeseed drying described in Section 2.2.2 were identified by XRD. Figure 11 shows the observed and calculated XRD patterns refined using the DDM method. The phase compositions of the studied samples are listed in Table 4 compared to the data on composition of the initial sample.

The MgSO_4_·H_2_O phase is most hydration-resistant and is observed in the samples until the second cycle. After the third sorption drying cycle, only the MgSO_4_∙7H_2_O (epsomite) phase is observed in the sample, which was absent after the first two cycles where the MgSO_4_∙6H_2_O phase was predominant.

**Figure 12:**
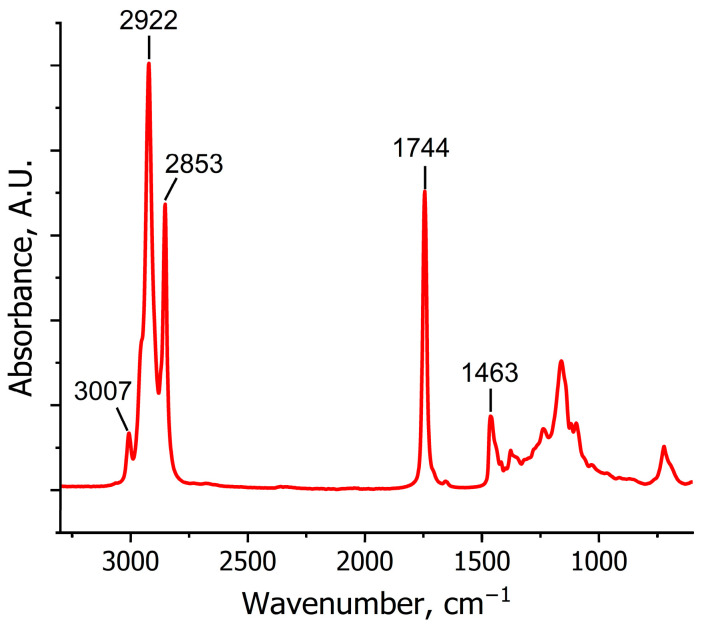
ATR-MIR spectra of rapeseed oil.

Oil integrity within rapeseed is an important criterion for choosing a drying method. In rapeseeds, oil is formed in oil bodies and, hypothetically, should not escape from the seeds during drying [50]. However, it was essential to assess whether oil is lost together with water during sorption drying. In the IR spectrum of rapeseed oil (Figure 12), the weak band at 3007 cm^−1^ corresponds to =C-H stretching vibrations of fatty acids. Two strong bands at 2922 cm^−1^ and 2853 cm^−1^ are ascribed to the asymmetric and symmetric vibrations of CH_2_ groups, respectively. In the fingerprint region, the highest-intensity band at 1744 cm^−1^ is ascribed to C=O stretching vibrations. Meanwhile, the band at 1700 cm^−1^, being characteristic of C=O stretching vibrations of carboxyl groups, is almost absent, indicating that fatty acids predominantly exist in their ester forms [51,52]. The spectrum also contains characteristic bands of CH_2_ scissoring vibrations (1463 cm^−1^) and symmetrical CH_3_ bending vibrations (1380 cm^−1^). Table 5 summarizes the bands observed in the IR spectrum of rapeseed oil.

**Figure 13:**
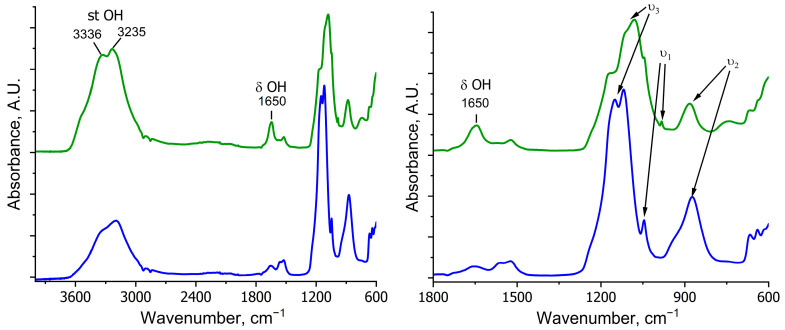
The ATR-MIR spectra of the initial desiccant (blue line) and after three-stage sorption drying of rapeseed (green line).

The IR spectrum of the desiccant (Figure 13) was interpreted based on the known characteristic bands of the sulfate group (T_d_ symmetry). Two strong bands at 1100 cm^−1^ and 1150 cm^−1^ can be ascribed to asymmetric stretching vibrations (ν_3_); the weak band at 870 cm^−1^ is assigned to bending vibrations (ν_2_). The weak band at 1020 cm^–1^ can be attributed to ν_1_(SO_4_), which is usually inactive in the IR spectrum but may appear because of slight symmetry distortions [53]. The spectrum of the initial desiccant shows no significant absorption in the 1500–2000 cm^−1^ range, where vibrations of adsorbed water would appear. However, a well-resolved band at 1650 cm^−1^, characteristic of bending vibrations of crystallization water, appears in the spectrum of the desiccant after drying [54]. Furthermore, a red shift is observed in the SO_4_ stretching bands, caused by symmetry distortion of the SO_4_^2−^ group, leading to band splitting (reduction of SO_4_ symmetry to C_3v_ or C_2v_ due to the influence of water) [53,55]. Meanwhile, the bending vibration band, being least sensitive to hydration, remains almost unshifted.

One can also see a significant rise in intensity of the broad band corresponding to OH stretching vibrations and water molecules in the post-drying sample compared to the initial sorbent. Notably, this broad signal consists of bands with two well-resolved peaks at 3336 and 3235 cm^−1^, both increasing in intensity upon water absorption. This phenomenon can be attributed to several factors characteristic of the kieserite structure:

- in the crystal structure of kieserite, water molecules can occupy non-equivalent positions such as coordinated water (Mg–OH_2_), with the band peaking at 3336 cm^−1^, and free or weakly bound water peaking at 3235 cm^−1^ [56];
- the presence of bending vibration bands of the OH group at 1650 cm^−1^ may lead to combination bands through Fermi resonance: the interaction between the fundamental OH vibration and the overtone of a bending mode. In this case, the two bands at 3336 and 3235 cm^−1^ could be a Fermi doublet [57,58].

Importantly, no other changes appear in the desiccant spectrum after rapeseed drying. The absence of new signals corresponding to rapeseed oil components confirms that oil remains confined within the seeds during sorption drying.

**Figure 14:**
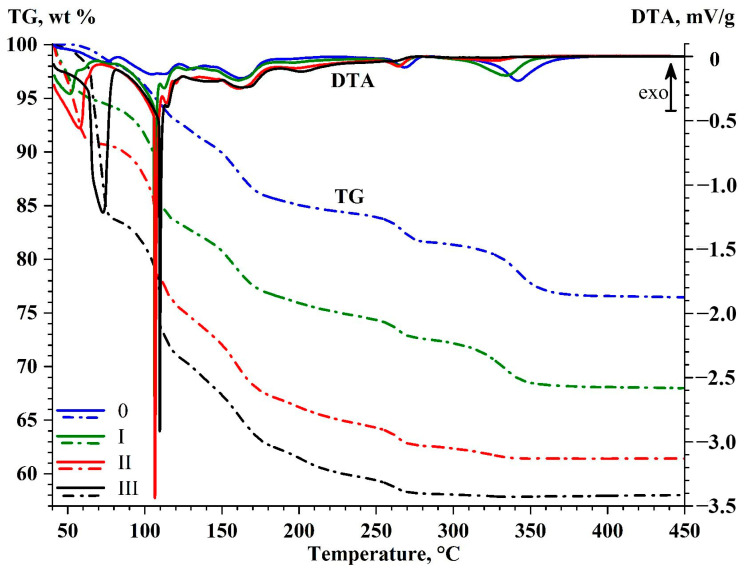
The DSC-TG-DTG curves of thermal transformation for the initial desiccant and after three-stage sorption drying of rapeseed.

Thermal analysis (DSC-TG) of magnesium sulfate samples after each of the three multi-stage cycles of rapeseed drying described in Section 2.2.2 was carried out in order to determine the amount of chemisorbed water and optimal regeneration temperatures. Assessment of synchronous thermal analysis data (Figure 14) revealed that the content of sorbed moisture increases when proceeding from the first drying cycle to the third one. This rise in the content of chemisorbed moisture in the desiccant manifests itself as increasing mass loss in the temperature range of 40–450 °C: 31.89, 38.52, and 41.99 wt%, respectively (Table 6), corresponding to the gross formulas of crystalline hydrates with the H_2_O/MgSO_4_ molar ratios equal to 3.13, 4.20, and 4.84, respectively.

A comparative analysis of the DSC curves of the initial desiccant and desiccant samples after three drying cycles (Figure 14) reveals several groups of endothermic peaks corresponding to the different stages of dehydration of MgSO_4_·*n*H_2_O crystalline hydrates, which make up the desiccant:

1. The low-temperature endothermic peaks with the extrema at 51–75 °C correspond to dehydration of MgSO_4_·*n*H_2_O crystalline hydrates (*n* ≥ 3) to a state approaching the metastable trihydrate MgSO_4_·3H_2_O.
2. The low-temperature peaks with extrema at 105–114 °C correspond to dehydration to a metastable state corresponding to sanderite MgSO_4_·2H_2_O [59,60]. There are abrupt endothermic peaks at 106, 107, and 110 °C, corresponding to the endothermic process of boiling saturated solution surrounding crystalline hydrate [59]. This crystalline hydrate was formed at the early stages of dehydration at 51–75 °C, when evaporation of free water not included in the crystalloid structure was thermodynamically constrained. Further loss of crystallization water (the weak peaks at 112–114 °C) gave rise to a mixture of metastable crystalline hydrates MgSO_4_·2H_2_O+ MgSO_4_·H_2_O [60].
3. The groups of peaks at 131–140 °C and 161–162 °C are associated with dehydration to a state approaching magnesium sulfate monohydrate MgSO_4_·H_2_O (kieserite). Notably, the formation of a stoichiometric monohydrate upon dehydration of crystalline hydrates MgSO_4_·*n*H_2_O (where *n* > 2) is extremely challenging [61] because of formation of an amorphous state with variable content of crystallization water (1.18–1.30 mole H_2_O per mole MgSO_4_), which is significantly affected by partial pressure of water vapor.
4. In the high-temperature region (above 185 °C), three groups of endothermic peaks are observed (°C): at 196–202, 263–268, and 325–342, being accompanied by mass loss equivalent to the loss of the final structural water molecule, resulting in formation of anhydrous MgSO_4_.

There is a trend toward greater contribution of low-temperature (below 185 °C) forms of crystallization water as the number of cycles of rapeseed sorption drying increases, with a corresponding decline in contribution of high-temperature forms up to the nearly complete disappearance of peaks above 185 °C (Figure 14).

The DSC-TG data suggest that the maximum regeneration temperature required to ensure desiccant generation to anhydrous magnesium sulfate after three-stage sorption drying of rapeseed decreases as one proceeds from the first stage to the third one (166, 158, and 154 °C, respectively) (Table 6). The optimal regeneration temperature range is 105–114 °C, corresponding to dehydration to the metastable state of sanderite MgSO_4_·2H_2_O.

## 3. Materials and Methods

### 3.1. Experimental Materials

The rapeseed (*Brássica nápus*) used in this study belonged to the Nadezhnyy 92 variety, which was produced in the East Siberian region (EPF “Mikhailovskoye”, Federal Research Center ‘Krasnoyarsk Scientific Center’ SB RAS, Krasnoyarsk, Russia) and was harvested in 2024.

Kieserite of fine crystalline grade, a commercial magnesium sulfate agrochemical, was used as a solid desiccant. This product was manufactured by the South Ural Plant of Magnesium Compounds (SUPMC, Kuvandyk, Orenburg Region, Russia). Kieserite was produced in compliance with the Technical Specifications TU 20.13.41-001-23877036-2017 [39]. The product underwent double drying, which allowed it to be stored for up to five years, and complied with both Russian and European quality standards. The agrochemical magnesium sulfate was formulated as a fertilizer containing mineral magnesium sulfur, intended to increase fertility and crop yield in all soil types [40]. According to the product certificate, the fine crystalline Kieserite magnesium sulfate contained at least 90 wt% MgSO_4_ and had a MgO content of at least 25.0 wt%. On a dry matter basis, the total mass fraction of impurity sulfates (calcium, sodium and iron) was ≤1 wt%, and the remaining volume of water-insoluble salts was ≤0.6 wt%.

### 3.2. Experimental Procedures

#### 3.2.1. Desiccant Characterization Methods

The physicochemical characteristics of the desiccant, including particle size distribution, the content of chemical components and individual crystalline phases, the number of crystallization water molecules, and regeneration temperature, were determined using particle size analysis, scanning electron microscopy–energy dispersive spectroscopy (SEM-EDS), X-ray diffraction analysis (XRD), attenuated total reflectance infrared spectroscopy (ATR-MIR), and simultaneous thermal analysis (DSC-TG).

The particle size distribution was determined using a VP-S/220 vibration drive (Vibrotechnic, St. Petersburg, Russia); the sieving duration was 60 min at a frequency of 70 Hz.

The SEM-EDS study was conducted using a TM-4000 scanning electron microscope (High Technologies Corporation, Hitachi, Tokyo, Japan) equipped with a Quantax 70 microanalysis system and a Bruker XFlash 430H energy-dispersive X-ray spectrometer (Bruker Corporation, Billerica, MA, USA) at a magnification of ×35–1800 and an accelerating voltage of 20 kV. Powder samples were applied to a double-coated conductive carbon adhesive tape (Ted Pella Inc., Altadena, CA, USA) attached to a flat substrate (1–3 mm thick, 30 mm in diameter) made of Duopur poly (methyl methacrylate) resin (Adler, Schwaz, Austria).

The phase composition was identified by quantitative X-ray powder diffraction analysis. The XRD data were obtained on an X’Pert Pro MPD powder diffractometer (PANalytical, Almelo, Netherlands) with a PIXcel solid-state detector using Cu Kα radiation. For minimizing the contact between the powder and atmospheric moisture, the samples were packed in a cuvette and sealed with a thin protective film. Two consecutive scans were made to control changes in the sample during shooting; their comparison revealed no noticeable changes. XRD patterns were recorded at room temperature in the range of diffraction angles 5° ≤ 2θ ≤ 70°. The quantitative phase composition was determined using the derivative difference minimization (DDM) method with normalization of the total amount of crystalline phases using the diffraction pattern of an external standard [62]. Sodium sulfate reagent Na_2_SO_4_ was used as an external standard. The phases present in the sample were identified using the ICDD PDF database [46].

The attenuated total reflectance infrared (ATR-IR) absorption spectra of the initial desiccant and desiccant after sorption drying of the rapeseed were recorded on an FTIR Spectrometer Vertex 70 (Bruker Optics GmbH & Co. KG, Ettlingen, Germany) equipped with a MIRacle ATR sampling accessory (PIKE Technologies, Madison, WI, USA), with a ZnSe monocrystal prism. Spectra were recorded in the range of 600–4000 cm^−1^ with a resolution of 4 cm^−1^ at room temperature.

Simultaneous thermal analysis (DSC–TG) was performed in a dynamic gas mixture of 20% O_2_ + 80% Ar with a total flow of 50 sccm, with simultaneous registration of mass changes and heat flow in a Jupiter STA 449C synchronous thermal analysis unit with an Aëolos QMS 403C mass spectral analyzer (Netzsch, Selb, Germany). Measurements were made in Pt-Rh crucibles with perforated lids at a linear temperature rate of 2.5 °C/min in the range of 40–450 °C with a sample weight of 11 ± 1 mg. The samples were crushed to a granulometric class of less than 160 μm; before starting heating, the samples were subjected to isothermal exposure in a flow of gas mixture for 5 min at 40 °C. The DSC sensor was heat flow calibrated by measuring the heat capacity of the sapphire disc according to the DIN 51007:1994-06 method [63]. Primary thermoanalytical data were processed using the licensed NETZSCH Proteus software package (Ver. 4.8.4) [63].

#### 3.2.2. Determination of Moisture Content

Hot air drying was used to determine the moisture content (MC) of the seeds; it was carried out in compliance with GOST 10856-1996 [64] at 130 ± 2 °C, which complied with the international standard ISO 665:2020 [65]. The moisture content was calculated on a wet basis (% wb). Statistical analysis of the data was carried out in accordance with GOST 10856-1996 [64], which determines the experimental data reproducibility. The allowable discrepancy between the results of two parallel determinations with a confidence probability of *p* = 0.95 should not exceed 0.25% abs. The average of the two parallel measurements is used as the final result.

The moisture content in the desiccant was determined based on the experimental weight loss data after heat treatment under complete dehydration conditions at 400 ± 2 °C.

#### 3.2.3. Sorption Drying Experiments

The experiments on sorption drying of rapeseed were carried out as follows. A batch of rapeseed with certain initial moisture content was placed into a closed container, and a desiccant was added. The moisture content in the desiccant was determined previously. The number of water molecules in the crystalline hydrate was calculated. The desiccant/rapeseed mass ratio was 1:2, 1:4, and 1:6. The container was placed on an automatic stirrer, and sorption drying was carried out for a specified time. The stirring frequency was 2 rpm. The temperature was measured using a resistance thermometer; the temperature sensing element was located in the center of the container with a rapeseed–desiccant mixture. After a given time, the rapeseed–desiccant mixture was placed on a sieve with a mesh size of 1 mm and separated. The moisture content in rapeseed was checked after 15, 30, 60, 90, 150, and 240 min after drying initiation.

For optimizing the drying process, the desiccant was used until it had been completely saturated with moisture without intermediate regeneration. Three batches of rapeseed were successively contact-dried. The desiccant/rapeseed mass ratio was 1:2. The sorption drying duration for each rapeseed batch was 240 min.

#### 3.2.4. Drying Kinetics

The loss of moisture in rapeseed during drying is time-dependent and can be represented by the moisture ratio (MR) profile. The initial moisture content in the rapeseed and moisture content at a given time during drying are required to perform this calculation. Seed moisture content is typically measured on a wet basis, which takes into account the mass of water per unit mass of wet seed [66].

The rapeseed drying curves were plotted using Equation (1) [17]. The kinetics of moisture adsorption by the desiccant during rapeseed drying are also described by Equation (1). The reproducibility of the empirical data and results was assessed using a relative error of determination not exceeding 10%. Statistical analysis of the experimental data was performed in accordance with GOST R ISO 5725-2-2002 [67] using the software Statistica, version 14.0.015 (StatSoft Europe GmbH, Hamburg, Germany). The following characteristics were considered such as mean values from selecting data of 9 for each data, standard error of measurement, and smallest real difference, etc.

#### 3.2.5. Rapeseed Germination Test

Rapeseed germination after sorption drying was assessed following a standard test according to the State Standard GOST 12038-84 [47] and the International Rules for Seed Testing [48]. The test was carried out in four replicates, each comprising 100 seeds, using the filter paper method at a temperature of 20 °C. The seed germination capacity was evaluated on day 7 of the experiment after planting the sample. Statistical data analysis was carried out in accordance with GOST 12038-84 [47], which determines the experimental data reproducibility. The arithmetic means of the results from analyzing all the samples is used as the final analysis result, provided that the deviations of individual sample results from the mean do not exceed certain thresholds when analyzing the germination rates of four parallel samples. With an arithmetic mean germination value between 92 and 94%, the allowable deviation of the analysis results for individual samples from the average should not exceed ±5%, between 88 and 91% it should not exceed ± 6%. The rapeseed qualities for planting were evaluated in accordance with the State Standard GOST 52325-2005 [49].

## 4. Conclusions

The study focused on sorption drying of rapeseed using commercial agrochemical magnesium sulfate MgSO_4_·*n*H_2_O (kieserite of fine crystalline grade) as a desiccant. The selected desiccant consists of 98% magnesium sulfate particles sized < 1.0 mm, ensuring that it is easily separated from rapeseed by sieving after drying. The moisture content in the desiccant is 24.9 wt%, corresponding to the composition of crystalline hydrate MgSO_4_·2.2H_2_O. Along with stable crystalline phases of kieserite MgSO_4_∙H_2_O and starkeyite MgSO_4_∙4H_2_O (28 wt% each), the desiccant contains 20 wt% of the metastable phase MgSO_4_∙2.5H_2_O.

A study of the kinetic features of sorption drying of rapeseed showed that the target moisture contents of ~7–8% are attained within 60–240 min of sorption drying depending on initial moisture content (12–16%) and the desiccant-to-rapeseed ratio (1:2, 1:4, 1:6). The study demonstrated the feasibility of reusing desiccant in multi-stage sorption drying of three rapeseed batches with initial moisture content of 12–14% and two batches with moisture content of 16 wt% at a desiccant-to-rapeseed mass ratio of 1:2. The MgSO_4_·H_2_O kieserite phase is most hydration-resistant, being retained in the samples until the second cycle. The MgSO_4_∙6H_2_O hexahydrate phase becomes predominant after two cycles, while the MgSO_4_∙7H_2_O epsomite phase is identified only after the third sorption drying cycle.

The DSC–TG data indicate that the optimal desiccant regeneration temperature is 105–114 °C, corresponding to dehydration to the metastable phase sanderite MgSO_4_·2H_2_O. ATR–FTIR analysis of the desiccant after sorption drying showed no signals characteristic of rapeseed oil, thus verifying that the oil component remains in seeds after dehydration using MgSO_4_·*n*H_2_O. Assessment of rapeseed germination after sorption drying showed that the seed germination capacity corresponds to that for elite rapeseed categories (ranging from 89 ± 1 to 92 ± 2%). Hence, the study of sorption drying of rapeseed using magnesium sulfate MgSO_4_·*n*H_2_O as a desiccant has demonstrated that this method is promising for reducing moisture content in oilseeds, while preserving their quality.

## Figures and Tables

**Table 1:** Moisture contents of rapeseeds and desiccant during sorption drying at various desiccant-to-rapeseed mass ratios and initial rapeseed moisture content.

Initial Rapeseed MC	Desiccant-to-Rapeseed Mass Ratio	Time (Min)						
		0	15	30	60	90	150	240
12%	Rapeseed moisture content (% wb) ± 0.1 (*p* = 0.95)							
	1:2	11.90	9.1	8.8	8.1	7.8	7.3	7.0
	1:4	11.90	9.6	9.0	8.4	8.0	7.5	7.1
	1:6	11.90	9.7	9.2	8.7	8.4	7.8	7.3
	Desiccant moisture content (wt%) ± 0.02 (*p* = 0.95)							
	1:2	25.19	27.38	28.28	29.48	30.35	31.40	32.18
	1:4	25.19	29.60	31.17	33.31	34.69	35.61	37.28
	1:6	25.19	30.79	33.22	35.77	37.49	39.06	40.75
14%	Rapeseed moisture content (% wb) ± 0.1 (*p* = 0.95)							
	1:2	13.8	11.6	10.6	9.4	8.9	8.0	7.3
	1:4	13.8	11.3	10.5	9.5	8.9	8.1	7.5
	1:6	13.8	11.6	10.7	9.8	9.2	8.6	8.2
	Desiccant moisture content (wt%) ± 0.02 (*p* = 0.95)							
	1:2	25.16	28.56	29.87	31.21	32.24	33.59	34.17
	1:4	25.16	30.81	33.65	36.29	37.55	39.15	40.46
	1:6	25.16	33.60	36.54	39.46	41.07	43.06	44.69
16%	Rapeseed moisture content (% wb) ± 0.1 (*p* = 0.95)							
	1:2	15.9	12.3	11.0	9.8	8.9	7.9	7.3
	1:4	15.9	12.5	11.3	10.3	9.4	8.5	7.9
	1:6	15.9	12.8	11.7	10.7	9.8	9.0	8.5
	Desiccant moisture content (wt%) ±0.02 (*p* = 0.95)							
	1:2	25.17	29.59	32.04	33.69	34.77	35.80	36.73
	1:4	25.17	33.38	36.26	39.31	40.79	42.06	43.71
	1:6	25.17	35.75	40.13	43.20	45.28	47.78	48.71

**Table 2:** The number of H_2_O molecules (*n*) in the MgSO_4_·*n*H_2_O crystalline hydrate at various desiccant-to-rapeseed mass ratios and initial rapeseed moisture content.

Initial Rapeseed MC	Desiccant-to-Rapeseed Grain Mass Ratio	Time (Min)						
		0	15	30	60	90	150	240
12%	Number of H_2_O molecules (*n*)							
	1:2	2.25	2.52	2.63	2.79	2.91	3.06	3.17
	1:4	2.25	2.81	3.03	3.34	3.55	3.69	3.97
	1:6	2.25	2.97	3.32	3.72	4.01	4.28	4.59
14%	1:2	2.25	2.67	2.85	3.03	3.18	3.38	3.47
	1:4	2.25	2.97	3.39	3.81	4.02	4.30	4.54
	1:6	2.25	3.38	3.85	4.35	4.66	5.05	5.40
16%	1:2	2.25	2.81	3.15	3.39	3.56	3.73	3.88
	1:4	2.25	3.35	3.80	4.33	4.60	4.85	5.19
	1:6	2.25	3.72	4.48	5.08	5.53	6.11	6.35

**Table 3:** The moisture content of rapeseed and desiccant and the number of *n*H_2_O molecules in the MgSO_4_·*n*H_2_O crystalline hydrate after three-stage sorption drying.

Stage	Rapeseed MC, wt% ±0.1 (*p* = 0.95)	Desiccant MC, wt% ±0.02 (*p* = 0.95)	Crystalline Hydrate Composition
MC_0_ of rapeseed~12%			
0	12.3	25.09	MgSO_4_·2.24H_2_O
I	6.9	32.40	MgSO_4_·3.20H_2_O
II	7.4	38.86	MgSO_4_·4.25H_2_O
III	7.9	44.20	MgSO_4_·5.29H_2_O
MC_0_ of rapeseed~14%			
0	13.8	25.09	MgSO_4_·2.24H_2_O
I	7.2	34.24	MgSO_4_·3.48H_2_O
II	7.6	41.30	MgSO_4_·4.70H_2_O
III	8.0	47.48	MgSO_4_·6.04H_2_O
MC_0_ of rapeseed~16%			
0	15.9	25.12	MgSO_4_·2.24H_2_O
I	7.2	36.74	MgSO_4_·3.88H_2_O
II	8.0	45.80	MgSO_4_·5.65H_2_O
III	10.2	50.66	MgSO_4_·6.86H_2_O

**Table 4:** Phase composition of the desiccant: 0—initial; I, II, III—after stage of contact drying (wt%).

Phase	Desiccant Sample			
	0	I	II	III
MgSO_4_·H_2_O	28(1)	21(1)	4.2(2)	–
MgSO_4_·2.5H_2_O	20.0(5)	– *	–	–
MgSO_4_·4H_2_O	28.2(7)	9.4(5)	–	–
MgSO_4_·6H_2_O	–	60.0(8)	94.7(8)	–
MgSO_4_·7H_2_O	–	–	–	83(1)
NaCl	0.8(1)	0.9(1)	0.7(1)	0.8(1)

*—not detected.

**Table 5:** Assignment of the peaks in the ATR-FTIR spectra of rapeseed oil.

Wavenumber, cm^−1^	Functional Group	Vibration Mode
3007	=CH	stretching
2922	C-H (CH_2_)	stretching asymmetrical
2853	C-H (CH_2_)	stretching symmetrical
1744	C=O	stretching
1463	C-H (CH_2_)	scissoring
1420	C-H	bending
1380	C-H (CH_3_)	symmetrical bending
1250–1100	C-O	stretching
873	CH_2_	rocking
690	HC=CH	bending out of plane

**Table 6:** Mass loss (∆m) and water capacity of desiccant samples subjected to three-stage sorption drying.

Desiccant Sample	Temperature Range, °C								(H_2_O)/(MgSO_4_), mol	
	40–100	40–150	40–200	40–250	40–300	40–350	40–400	40–450		
	∆m, wt%									
0	3.78	10.07	14.93	16.06	18.62	22.21	23.42	23.53	2.06	
I	8.25	19.11	23.95	25.53	27.71	31.39	31.78	31.89	3.13	
II	12.28	27.87	33.72	35.64	37.58	38.51	38.52	38.52	4.20	
III	18.77	32.64	38.52	40.61	41.94	42.14	42.05	41.99	4.84	
	Water capacity, mg/g anhydrous *								∆m, wt% **	T, °C ***
0	49	132	195	210	243	290	306	308		
I	121	281	352	375	407	461	467	468	21.70	165.5
II	200	453	548	580	611	626	627	627	29.32	157.6
III	324	563	664	700	723	726	725	724	33.31	154.2

*—values are given per gram of anhydrous composition at 450 °C; **—∆m to final state MgSO_4_∙H_2_O; ***—initial temperature of H_2_O loss in MgSO_4_∙H_2_O.

## Data Availability

The data presented in this study are available on request from the corresponding author.

## Abbreviations

The following abbreviations are used in this manuscript:

ATR-MIR	Attenuated total reflectance infrared spectroscopy
DSC-TG	Differential scanning calorimeter—Thermogravimetry
EDS	Energy-dispersive spectroscopy
MC	Moisture content
MR	Moisture ratio
SEM	Scanning electron microscopy
XRD	X-ray diffraction
